# Supplementary material for: Performance Evaluation of the IR Biotyper® System for Clinical Microbiology: Application for Detection of Staphylococcus aureus Sequence Type 8 Strains
Source: Antibiotics (Basel). 2022 Jul 7;11(7):909. doi: 10.3390/antibiotics11070909 (PMC9311605; doi:10.3390/antibiotics11070909)
Supplement: Supplementary file 1 [file antibiotics-11-00909-s001.zip › antibiotics-1786844-supplementary.pdf]

Table S1. The information of the primers used in this study

Target	Primer name	Primer sequence	Annealing Tm	Amplicon size	Reference
mecA	mecA-P4	TCCAGATTACAACTTCACCAGG	59℃	162	1
	mecA-P7	CCACTTCATATCTTGTAACG			
mecC	mecC MFP	GAAAAAAAGGCTTAGAACGCCTC	59℃	138	2
	mecC MRP	GAAGATCTTTTCCGTTTTCAGC			
pvl	PVL-F	ATCATTAGGTAAAATGTCTGGACATGATCCA	55℃	433	3
	PVL-R	GCATCAASTGTATTGGATAGCAAAAGC			
tst-1	tsst-F	ATGGCAGCATCAGCTTGATA	55℃	350	4
	tsst-R	TTTCCAATAACCACCCGTTT			
SCC*mec* typing	CIF2F2	TTCGAGTTGCTGATGAAGAAGG	50℃	495	1
	CIF2R2	ATTTACCACAAGGACTACCAGC			
	KDPF1	AATCATCTGCCATTGGTGATGC		284	
	KDPR1	CGAATGAAGTGAAAGAAAGTGG			
	MECIP2	ATCAAGACTTGCATTCAGGC		209	
	MECIP3	GCGGTTTCAATTCACTTGTC			
	SCCmec III J1-F	CATTTGTGAAACACAGTACG		243	
	SCCmec III J1-R	GTTATTGAGACTCCTAAAGC			
	RIF5F10	ATATACCAAACCCGACAACTACA		414	
	RIF5R13	CATAACTTCCCATTCTGCAGATG			
	ccrB2-F2	AGTTTCTCAGAATTCGAACG		311	
	ccrB2-R2	CCGATATAGAAWGGGTTAGC			
	ccrC-F2	GTACTCGTTACAATGTTTGG		449	
	ccrC-R2	ATAATGGCTTCATGCTTACC			
	SCCmec V J1-F	TTCTCCATTCTTGTTCATCC		377	
	SCCmec V J1-R	AGAGACTACTGACTTAAGTGG			
	dcs-F2	CATCCTATGATAGCTTGGTC		342	
	dcs-R1	CTAAATCATAGCCATGACCG			
	MR1_mecA-F	ATGAGATTAGGCATCGTTCC		554	
	MR2_mecA-R	TGGATGACAGTACCTGAGCC			
spa type	1095F	AGACGATCCTTCGGTGAGC	58℃	Variable	5
	1517R	GCTTTTGCAATGTCATTTACTG			

A

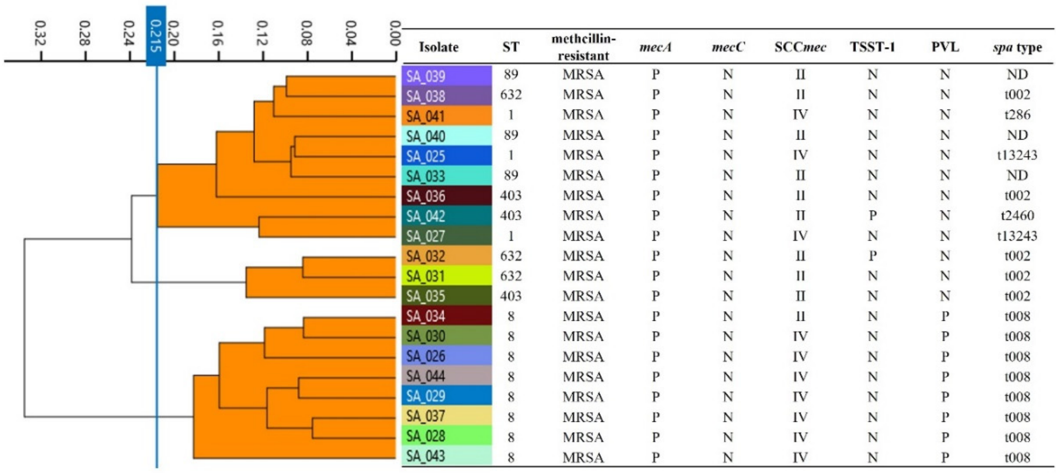

Isolate	ST	methcillin-resistant	*mecA*	*mecC*	SCC*mec*	TSST-1	PVL	*spa* type
SA_039	89	MRSA	P	N	II	N	N	ND
SA_038	632	MRSA	P	N	II	N	N	t002
SA_041	1	MRSA	P	N	IV	N	N	t286
SA_040	89	MRSA	P	N	II	N	N	ND
SA_025	1	MRSA	P	N	IV	N	N	t13243
SA_033	89	MRSA	P	N	II	N	N	ND
SA_036	403	MRSA	P	N	II	N	N	t002
SA_042	403	MRSA	P	N	II	P	N	t2460
SA_027	1	MRSA	P	N	IV	N	N	t13243
SA_032	632	MRSA	P	N	II	P	N	t002
SA_031	632	MRSA	P	N	II	N	N	t002
SA_035	403	MRSA	P	N	II	N	N	t002
SA_034	8	MRSA	P	N	II	N	P	t008
SA_030	8	MRSA	P	N	IV	N	P	t008
SA_026	8	MRSA	P	N	IV	N	P	t008
SA_044	8	MRSA	P	N	IV	N	P	t008
SA_029	8	MRSA	P	N	IV	N	P	t008
SA_037	8	MRSA	P	N	IV	N	P	t008
SA_028	8	MRSA	P	N	IV	N	P	t008
SA_043	8	MRSA	P	N	IV	N	P	t008

B

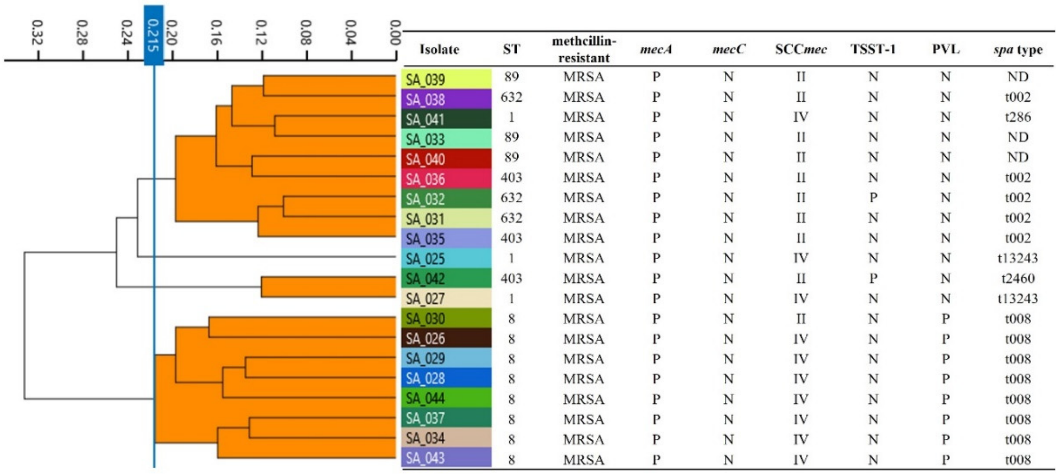

Isolate	ST	methcillin-resistant	*mecA*	*mecC*	SCC*mec*	TSST-1	PVL	*spa* type
SA_039	89	MRSA	P	N	II	N	N	ND
SA_038	632	MRSA	P	N	II	N	N	t002
SA_041	1	MRSA	P	N	IV	N	N	t286
SA_033	89	MRSA	P	N	II	N	N	ND
SA_040	89	MRSA	P	N	II	N	N	ND
SA_036	403	MRSA	P	N	II	N	N	t002
SA_032	632	MRSA	P	N	II	P	N	t002
SA_031	632	MRSA	P	N	II	N	N	t002
SA_035	403	MRSA	P	N	II	N	N	t002
SA_025	1	MRSA	P	N	IV	N	N	t13243
SA_042	403	MRSA	P	N	II	P	N	t2460
SA_027	1	MRSA	P	N	IV	N	N	t13243
SA_030	8	MRSA	P	N	II	N	P	t008
SA_026	8	MRSA	P	N	IV	N	P	t008
SA_029	8	MRSA	P	N	IV	N	P	t008
SA_028	8	MRSA	P	N	IV	N	P	t008
SA_044	8	MRSA	P	N	IV	N	P	t008
SA_037	8	MRSA	P	N	IV	N	P	t008
SA_034	8	MRSA	P	N	IV	N	P	t008
SA_043	8	MRSA	P	N	IV	N	P	t008

C

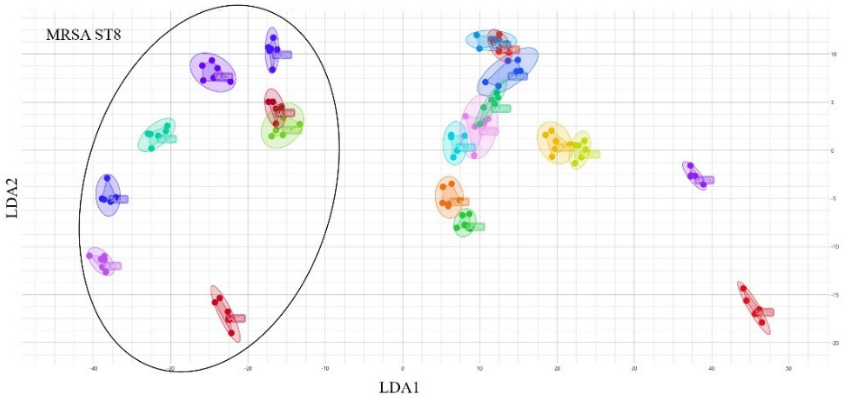

Figure S1. Dendrograms and 2D scatter plot of 20 S. aureus isolates based on the IRBT® spectra on TSA medium at cut-off of 0.215 with bacterial molecular profiles. A; 20 spectra of S. aureus isolates on TSA medium by averaging three technical replicates on the first day, B; 20 spectra of S. aureus isolates on TSA medium by averaging three technical replicates on the second day, C; Assessment of 2D scatter plot for 60 spectra of LDA analyses on TSA medium. MRSA ST8 isolates are successfully separated from MRSA ST1, ST89, ST403, and ST632. Abbreviation: ND, not-determined.

Reference

1. Duarte C.; Oliveira, Hermínia de Lencastre. Multiplex PCR Strategy for Rapid Identification of Structural Types and Variants of the mec Element in Methicillin-Resistant Staphylococcus aureus. *Antimicrobial Agents and Chemotherapy* **2002**, *46*, 2155-2161.

2. Stegger M.; Andersen PS.; Kearns A.; Pichon B.; Holmes MA.; Edwards G.; Laurent F.; Teale C.; Skov R.; Larsen AR. Rapid detection, differentiation and typing of methicillin-resistant Staphylococcus aureus harbouring either mecA or the new mecA homologue mecA(LGA251). *Clin Microbiol Infect* **2012**, *18*, 395-400.

3. Lina G.; Piémont Y.; Godail-Gamot F.; Bes M.; Peter MO.; Gauduchon V.; Vandenesch F.; Etienne J. Involvement of Panton-Valentine leukocidin-producing Staphylococcus aureus in primary skin infections and pneumonia. *Clin Infect Dis* **1999**, *29*,1128-1132.

4. Johnson WM.; Tyler SD.; Ewan EP.; Ashton FE.; Pollard DR.; Rozee KR. Detection of genes for enterotoxins, exfoliative toxins, and toxic shock syndrome toxin 1 in Staphylococcus aureus by the polymerase chain reaction. *J Clin Microbiol* **1991**, *29*, 426-430.

5. Harmsen D.; Claus H.; Witte W.; Rothgänger J.; Claus H.; Turnwald D.; Vogel U. Typing of methicillin-resistant Staphylococcus aureus in a university hospital setting by using novel software for spa repeat determination and database management. *J Clin Microbiol* **2003**, *41*, 5442-5448.
